# Early Postoperative Gait Analysis in Elderly Patients Following Hip Fracture Surgery

**DOI:** 10.3390/s25061888

**Published:** 2025-03-18

**Authors:** Gereon Anton Hecht, Rachel Senden, Rik Marcellis, Matthias Mertes, Paul Willems, Kenneth Meijer, Martijn Poeze, Taco J. Blokhuis

**Affiliations:** 1 Department of Trauma Surgery, Maastricht University Medical Centre (MUMC+), 6229 HX Maastricht, The Netherlands; 2 Department of Physical Therapy, Maastricht University Medical Centre (MUMC+), 6229 HX Maastricht, The Netherlands; 3 Department of Nutrition and Movement Sciences, Maastricht University, 6211 LK Maastricht, The Netherlands; 4 School of Nutrition and Translational Research in Metabolism (NUTRIM), Maastricht University, 6211 LK Maastricht, The Netherlands

**Keywords:** hip fractures, gait analysis, biofeedback, postoperative care

## Abstract

Hip fractures in elderly patients significantly reduce independence and quality of life. Early postoperative gait performance remains poorly understood, particularly regarding differences between surgical treatments, such as proximal femur nailing and hemiarthroplasty. Identifying gait alterations early in rehabilitation could optimize clinical interventions. This prospective observational cohort study included 40 elderly patients hospitalized after acute hip fracture surgery. Relative peak force and step duration were assessed using the ambulant pressure biofeedback system during postoperative mobilization. Additionally, three-dimensional gait analysis evaluated spatiotemporal parameters and sagittal plane kinematics of the hip, knee, and ankle. Results demonstrated significant improvements in median peak force (45.32% to 70.00%, (*p* < 0.001)) and median step duration (2.96 s to 137 s, (*p* < 0.001)) at the end of the hospitalization period. No significant differences in step duration and peak force were observed between the different surgical procedures, proximal femur nail, and hemiarthroplasty. Three-dimensional gait analysis showed significantly reduced hip extension during terminal stance in the operated leg compared to the healthy leg. These findings highlight the utility of biofeedback systems for monitoring early rehabilitation progress and emphasize the importance of 3D gait analysis in identifying early postoperative gait deficits. Targeted interventions during hospitalization could enhance functional recovery and improve patient outcomes.

## 1. Introduction

Hip fractures in elderly patients lead to a significant decline in independence and a reduced quality of life (QoL) [1]. Adequate postoperative management is crucial to regain functionality and QoL [2,3,4,5]. The early rehabilitation regimen after hip fracture surgery consists of physiotherapy focusing on restoring independent activities of daily living, including walking [6]. Research has shown that early mechanical loading after a fracture positively impacts bone density, fracture healing, functional recovery, and QoL [7,8,9,10,11,12]. However, translating these findings into meaningful gait measurements remains challenging. Research on the early progression of walking performance, particularly regarding step duration and weight-bearing on the operated leg, is still lacking—mainly due to the absence of reliable measurement instruments.

A potential solution to this issue is real-time weight-bearing detection, which is increasingly being integrated into clinical practice. In this context, biofeedback systems provide a feasible and clinically validated approach, offering real-time biofeedback on key parameters, such as step duration and weight-bearing, to both patients and therapists [13,14].

A rehabilitation center-based study by Raaben et al. observed substantial variations in mean relative peak forces and step durations of the operated leg in a cohort of geriatric patients (n = 113), starting 14 days post hip fracture surgery, measured using the biofeedback system “SensiStep” [13,15]. To categorize these variations and assess rehabilitation progress, four quadrants were defined based on mean relative peak force and mean step duration during rehabilitation following hospital discharge. These quadrants ranged from quadrant one (high relative peak force, short step duration) to quadrant four (low relative peak force, long step duration). The study found that patients progressively shifted from quadrant four to quadrant one over the rehabilitation period, indicating significant rehabilitation progress, ultimately resulting in near-normal values [15].

However, there is a lack of data on rehabilitation progress in terms of relative peak force and step duration during the first 14 days post hip fracture surgery while patients are still hospitalized. Collecting such data could facilitate the development of tailored rehabilitation protocols, potentially optimizing postoperative treatment.

Proximal femur fractures are typically classified into two categories: intracapsular fractures, which involve the femoral neck, and extracapsular fractures, which occur within the trochanteric region of the femur [1,16]. In geriatric patients, hip arthroplasty (hemiarthroplasty) is the most common surgical treatment for intracapsular fractures, whereas proximal femur nailing is typically used for extracapsular fractures due to their effectiveness across various fracture types and the relatively straightforward nature of these procedures [1]. Foss et al. observed that post surgical pain varies depending on the surgical procedure. Patients treated with a proximal femur nail reported higher pain scores compared to those who underwent hip arthroplasty, as measured using the verbal ranking score [17]. However, it remains unclear whether the two surgical procedures—hip arthroplasty and proximal femur nailing—differ in their effects on gait and its progression, particularly in terms of relative peak force and step duration.

A detailed examination of postoperative walking performance can be achieved through 3D gait analysis, a technique proven to be both reproducible and valid across various patient populations [18]. However, 3D gait analysis has not been extensively applied to frail patients immediately after hip fracture surgery due to its complexity, as well as postoperative fatigue, pain, and compliance challenges in elderly patients [19,20,21]. Conducting a 3D gait analysis shortly after hip fracture surgery could provide valuable insights into early postoperative walking performance, including spatiotemporal parameters and joint kinematics. This information could help guide targeted physical therapy interventions during hospitalization.

The primary aim of this study is to investigate whether step duration and relative peak force improve during the hospital stay in elderly patients following hip fracture surgery, by comparing biofeedback measurements taken as soon as possible after surgery with those recorded at the end of hospitalization. Additionally, we aim to explore whether gait progression, as measured by step duration and relative peak force, differs between the two common surgical procedures—proximal femur nailing and hemiarthroplasty. Lastly, 3D gait analysis will be used to assess spatiotemporal parameters and sagittal joint kinematics of both the operated and non-operated leg, providing comprehensive insights into early postoperative walking performance in this patient population.

## 2. Materials and Methods

### 2.1. Study Population

This prospective observational cohort study included patients with proximal femur fractures who underwent surgical intervention between April 2021 and July 2022 at the Department of Trauma Surgery at Maastricht University Medical Centre (MUMC+). Surgical treatment was determined according to the Dutch guidelines for the management of proximal femur fractures [22].

The following eligibility criteria were applied for study participation: proximal femur fracture resulting from low-energy trauma (fall from a standing position), age > 60 years, and body weight <120 kg to ensure sensor accuracy and permit unrestricted weight-bearing following surgical treatment. Patients with pre-existing comorbidities affecting gait and cognitive impairments, measured using the Mini-Mental State Examination (MMSE; <18 points), were excluded [23].

The following patient characteristics were recorded: gender (female/male), age (years), body height (cm), body weight (kg), and body mass index (BMI; kg/m^2^). Additionally, data were collected on the Almelo Hip Fracture Score (AHFS), MMSE, length of hospital stay (days), days from surgery until the first SensiStep measurement, surgical procedure following hip fracture (proximal femur nailing, sliding/dynamic hip screw, hemiarthroplasty, total hip replacement, and femoral neck system), and comorbidities (cardiovascular, pneumological, obesity, diabetes mellitus, and multimorbidity) [24,25].

Power analysis indicated a required sample size of 37 participants, based on α = 0.05, β = 0.8, an anticipated 10% loss to follow-up, and a medium effect size (Cohen’s d = 0.5).

### 2.2. Study Protocol

#### Biofeedback Measurements

**Figure 1:**
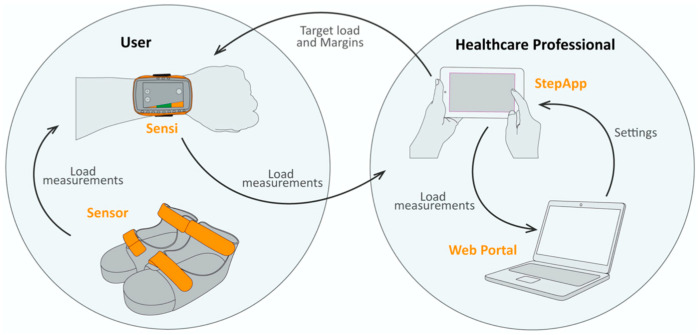
Representation of the biofeedback system, the “SensiStep”, with the different parts [13].

As soon as possible after surgery, patients started mobilization once a day under the supervision of a physiotherapist following the institutional protocol for the elderly after hip fracture surgery. During these physiotherapy sessions, the patients were wearing the SensiStep (Evalan BV, Amsterdam, the Netherlands) sandal on both feet (Figure 1) [13]. One researcher (G.H.) applied the sandals, dependent on foot size, for every measurement session. The sandal includes a pressure sensor under the midfoot of the operated leg, which registers pressure loads during every foot contact with the ground with a sampling rate of 50 Hz. Simultaneously, a coupled time stamp registers the corresponding time in milliseconds to determine step duration [13]. Daily mobilization was facilitated using walking aids, including 5.5 m parallel bars, wheeled or non-wheeled walkers, and crutches, depending on the patient’s specific needs. During the session, neither the patients nor the supervising physiotherapist received real-time biofeedback from the SensiStep; this was to prevent bias by (unconscious) adjustments of the gait pattern. Raw data were stored on a secured webportal and later downloaded for data processing and analysis.

Specific Matlab routines (Matlab R2014a, MathWorks), based on published principles, were used to calculate parameters of interest: (1) relative peak force (%), is defined as the maximum peak lead during the entire gait cycle of a step, relative to the patient’s bodyweight and (2) step duration (s) defined as the time between two successive peak forces of the same foot [13,26,27]. These parameters were determined for every recorded step of the operated leg. Steps with a step duration exceeding 5 s were considered outliers and excluded from analysis. For the first and the last session, the mean relative peak force (%) and mean step duration (s) were calculated for every patient. Finally, the medians over the groups (first vs. last session and proximal femur nail vs. hemiarthroplasty) were calculated. Furthermore, every step of every patient was categorized into quadrants according to pre-defined values. This was performed for the first and last SensiStep measurement. Quadrants were defined as followed: (1) high relative peak force and high step duration; (2) high relative peak force and low step duration; (3) low relative peak force and high step duration; and (4) low relative peak force and low step duration [15].

### 2.3. 3D Gait Analysis

A subgroup of the study population underwent an overground 3D gait analysis in the movement laboratory of the MUMC+ following the protocol for 3D gait analysis using the overground approach [28]. In short, patients had to walk at least five times over a 10 m walkway with an integrated force plate (1000 Hz, AMTI Biomechanics Force Platform model OR6-7) at a comfortable walking speed while 8 infrared cameras (100 Hz, Vicon T10S Motion System, Nexus 1.0, Oxford, UK) detected the reflecting markers which were attached to the whole patient’s body according to the Plug-In Gait (PiG) model [29]. During walking, patients were allowed to use a walking aid.

Data were processed in Nexus (V1.8.5) and custom-made algorithms, programmed in Matlab (R2016a, Mathworks, Natick, MA, USA), were used to check the quality of data, to identify steps, and to calculate gait parameters. Spatiotemporal parameters, such as walking speed (m/s), cadans (steps per minute), stance time (s), and swing time (s), were calculated. Additionally, sagittal joint kinematics of the hip, knee, and ankle were determined for every single step. Joint angles were normalized for time with 0% representing initial contact and 100% representing almost the next initial contact of the same leg. For every patient, averages over all valid strides were calculated and finally, group averages (first vs. last session and proximal femur nail vs. hemiarthroplasty) were determined.

### 2.4. Statistical Analysis

Normality of data was tested using the Shapiro Wilk test. Continuous data were expressed as median and interquartile range (IQR), absolute numbers (n), or percentages (%). Depending on the normality of data, a paired sample *t*-test or Wilcoxon signed rank test was used to compare the relative peak force and step duration between the first and last SensiStep measurement representing the start of mobilization with the physiotherapist and the end of the hospital stay. An independent sample t-test or Mann–Whitney U test was used to compare baseline characteristics, peak force, and step duration between the proximal femur nail and hemiarthroplasty surgery groups. Statistical parametric mapping (SPM) paired t-test was used to compare sagittal kinematic waveforms of the hip, knee, and ankle between the healthy and operated leg. Kinematic waveforms were presented as group averages with standard deviations. SPM analyses were implemented using the open source spm1d (www.spm1d.org (accessed on 8 July 2024)) in MATLAB (Mathworks, R2016a). The significance level was set at *p* < 0.05 for all analyses. All data were analyzed using SPSS (IBM statistics version 25). A *p*-value < 0.05 was considered statistically significant.

## 3. Results

### 3.1. Participants

In this study, 144 patients were assessed for eligibility, of which 104 were excluded: nine patients refused to participate, nine were ≤60 years of age, two exceeded a body weight of 120 kg, two were not allowed partial weight-bearing, 18 had severe gait comorbidities, one had a hip fracture due to high-energy trauma, 19 had cognitive impairments (MMSE < 18), six had pathological fractures, 16 underwent conservative treatment after hip fracture, 16 had inadequate mobilization prior to the first measurement, three had a hospital stay of less than one week, and three had a SARS-CoV-2 infection. In total, 40 patients were included in this study for SensiStep measurements. Among these, 29 patients were categorized into the two common surgical procedure groups: proximal femur nail (n = 19) and hemiarthroplasty (n = 10). Patients treated with a hemiarthroplasty had a significantly higher bodyweight in comparison to the patients treated with an intramedullary femur nail (*p* = 0.03). No other differences were found. Nine of all patients underwent an additional 3D gait analysis. Patient characteristics are presented in Table 1.

### 3.2. Biofeedback Measurements During Hospitalization

**Figure 2:**
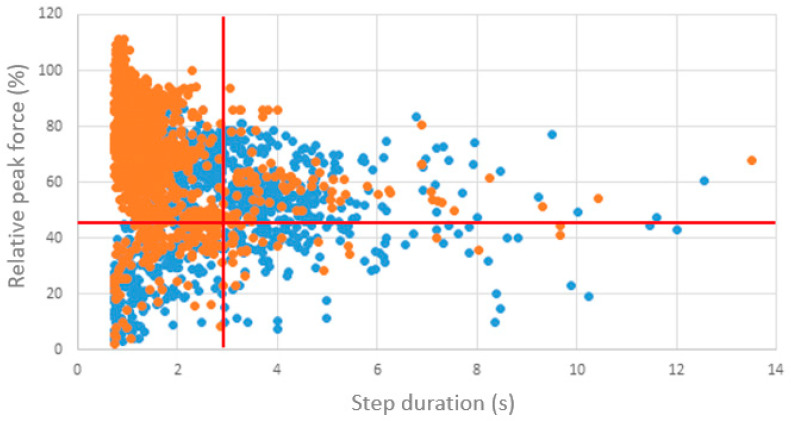
Step duration (s) and relative peak force (%) measured of all steps of all patients during the first (blue) and last (orange) session. The red line represents the median of step duration and peak force of all patients of the first measurement session, creating 4 quadrants as used in the study conducted by Raaben et al. Quadrant 1 (Q1): high relative peak force and short step duration. Q2: high relative peak force and long step duration. Q3: low relative peak force and short step duration. Q4: low relative peak force and long step duration.

During the first SensiStep measurement, the median (IQR) relative peak force and step duration were 45.32% (25.49%) and 2.96 s (1.17 s), respectively. During the last session, the median relative peak force was significantly (*p* < 0.001) increased to 70.00% (32.14%), while the median step duration was significantly (*p* < 0.01) decreased to 1.37 s (1.21 s) compared to the first session. Large interquartile ranges were observed during the first and last session demonstrating large variability among patients. The relative peak forces and step durations of all recorded steps measured during the first and last sessions are presented in four quadrants in Figure 1. In the first session, variation was substantial with steps reaching all four quadrants. A shift towards Q1, representing high relative peak force and short step duration, was observed during the last session (Figure 2).

**Figure 3:**
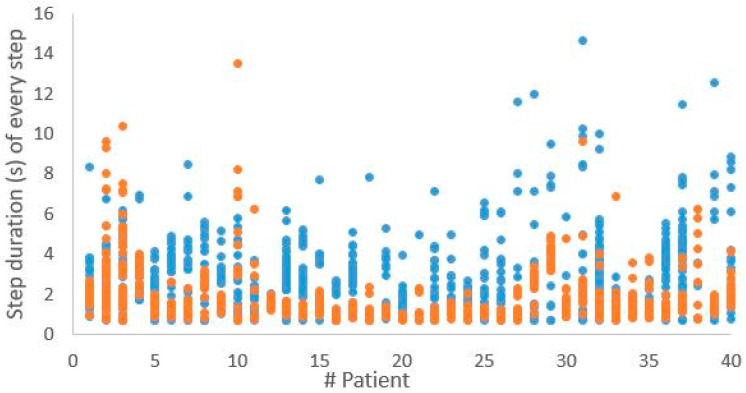
The step duration of all measured steps per patient, with the first session represented in blue and the last session represented in orange.

**Figure 4:**
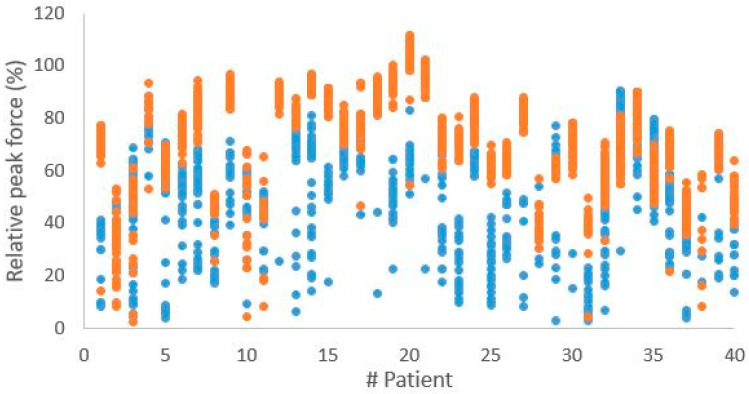
The step relative peak force of all measured steps per patient, with the first session represented in blue and the last session represented in orange.

The change towards steps with higher relative peak forces and shorter durations is observed in almost every patient, as is shown in Figure 3 and Figure 4, which present the relative peak force and step duration of every single step of every patient for both the first and last session.

### 3.3. Surgical Procedures and Biofeedback Measurements

Nineteen patients had undergone proximal femur nail surgery, and ten patients were treated with hemiarthroplasty due to proximal femur fractures (Table 1). Bodyweight was significantly higher in patients with a hemiarthroplasty (77.00 kg (26.75 kg)) compared to those with a proximal femur fracture (65.00 kg (15.00 kg); *p* = 0.033). The groups were comparable to the other baseline characteristics. For both, the proximal femur nail and the hemiarthroplasty groups, the relative peak force significantly increased between the first and last session (37.50% (34.17%) vs. 70.22% (35.60%), *p* < 0.001 and 46.44% (24.57%) vs. 69.19% (48.21%), *p* = 0.024). Step duration decreased from the first to the last session, showing a significant decrease for the proximal femur nail group (3.01 s (0.96 s) vs. 1.31 s (1.49 s), *p* < 0.001) and a non-significant decrease for the hemiarthroplasty group (2.72 s (1.09 s) vs. 1.50 s (1.64 s), *p* = 0.114).

### 3.4. Three-Dimensional Gait Analysis: Operated Versus Healthy Leg

The 3D gait analysis was performed median (IQR) 6.00 (1.00) days after surgery. The median (IQR) walking speed and cadence were, respectively, 0.40 (0.17) m/s and 61.21 (11.85) steps/min. Stance time was significantly shorter in the operated leg compared to the healthy leg (median (IQR) 1.38 (0.35) s vs. 1.44 (0.32) s, (*p* = 0.011)). Swing time was significantly longer in the operated leg compared to the healthy leg (median (IQR) 0.54 (0.06) s vs. 0.50 (0.10) s, *p* = 0.011). Sagittal hip kinematics of the operated leg were significantly different from the healthy leg, showing a lack of hip extension in the operated leg during the terminal stance phase (40–78% of gait cycle).

**Figure 5:**
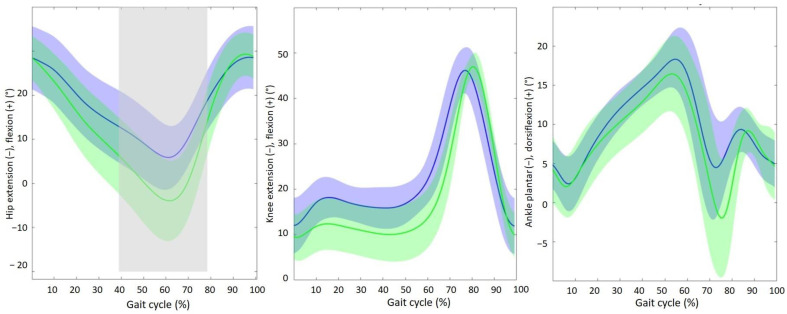
Averaged (SD) sagittal joint kinematics of the operated (blue) and healthy (green) leg for the hip, knee, and ankle as a function of the gait cycle (0–100%). The gray shaded areas indicate significant differences in averaged sagittal joint kinematics between the operated and healthy leg.

The mean difference in maximal hip extension was 9.82° (mean (SD) operated leg 5.91° (7.69°) vs. healthy leg −3.91° (9.76°)) (Figure 5). Sagittal knee and ankle angles of the operated leg did not differ significantly compared to the healthy leg, although the operated leg showed increased knee flexion during stance (e.g., at 50% of the gait cycle, mean (SD) operated leg 16.57° (4.71°) vs. healthy leg 10.35° (6.55°)) and reduced plantar flexion during push off (e.g., at 75% of the gait cycle, mean (SD) plantar flexion operated leg 4.64° (6.18°) vs. healthy −1.60° (8.30°)) compared to the healthy leg.

## 4. Discussion

This study demonstrated a significant increase in median relative peak force from 45.32% (IQR 25.49%) during the first measurement to 70.00% (IQR 32.14%) in the last measurement, while the median step duration decreased from 2.96 s (IQR 1.17 s) to 1.37 s (IQR 1.21 s). No significant differences in step duration or peak force were observed between the surgical procedures (intramedullary femur nail vs. hemiarthroplasty). Additionally, 3D gait analysis revealed a mean difference of 9.82° in maximal hip extension between the operated leg and the healthy leg.

This study is the first to explore gait in elderly hip fracture patients at an early stage after surgery. It demonstrates that gait, measured by an ambulatory pressure sensor, significantly improves at an early stage in terms of step duration and relative peak force during hospitalization following hip fracture surgery.

Previous research has established the use of pressure sensor technology to measure rehabilitation progress in the rehabilitation context [13,14,15,30]. However, no information existed on early clinical gait performance during hospitalization after hip surgery. This is the first study addressing this gap by demonstrating that it can objectively measure gait performance during hospital stay following hip fracture surgery. Patients improved during hospitalization, as shown by a significant reduced step duration and increased relative peak force from the first to the last measurement session. This progression in gait was confirmed in the quadrant analysis, which is in agreement with Raaben et al. [15]. However, the median relative peak force and the median step duration in our study was, respectively, lower and higher than found by Raaben et al. who measured during rehabilitation after discharge. This indicates that the gait of patients after hip fracture surgery further improves after discharge from the hospital. In addition, large interquartile ranges in step duration and relative peak force were found during the measurements, indicating large individual variation in these gait parameters. This shows that the impact of the surgery is different for each patient, resulting in gait differences at the start of the clinical rehabilitation. This study also showed that patients improve in gait during the early phase after surgery. This progress may be influenced by patient-specific factors, such as pain, fear of pain, anxiety, or muscular imbalances. Identifying these factors allows for the implementation of targeted early rehabilitation strategies tailored to individual patients. In addition, real-time measurement with a wearable pressure device, such as SensiStep, allows the treating physician to support the patient effectively during mobilization, and facilitates the implementation of tailor-made rehabilitation strategies.

The significant increase in relative peak force in both patient groups treated with a proximal femur nail or a hemiarthroplasty, as well as the reduction in step duration, demonstrates that rehabilitation shortly after surgery is effective, regardless of the surgical procedure. The lower body weight in the proximal femur nail group might explain the better functional recovery and faster improvement in step duration. Additionally, the proximal femur nail is a technically less invasive procedure with minimal soft tissue damage and, therefore, results in less functional loss as less muscle and soft tissue are damaged during surgery. The lack of significance in the reduction in step duration in the hemiarthroplasty group could be attributed to the smaller sample size or the differing biomechanical loads imposed by the implant [17].

In our study, nine patients underwent a detailed 3D gait analysis with a median (IQR) age of 77.00 (8.50) years, and median (IQR) 6.00 (1.00) days after hip fracture surgery. Previous gait analysis studies focused on gait in patients following hip joint replacement due to osteoarthritis instead of hip fractures. However, this is the first study performed during hospital stay in a frail and older population early after hip surgery. Nevertheless, in line with the existing body of literature, a study by Colgan et al. demonstrated a significantly shorter stance phase of the operated leg compared to the healthy leg following total hip replacement surgery (59.84% vs. 62.24%, *p* = 0.02) [31]. Regarding kinematic data, we found that the operated leg remains in flexion during terminal stance, where hip extension is expected. Bahr et al. demonstrated a reduced hip extension in the operated leg compared to the contralateral healthy leg (1.20° ± 11.10° vs. −7.76° ± 4.10°; *p* = 0.0001) at 16.7 months after total hip arthroplasty [32]. This suggests that improving hip extension in early rehabilitation could lead to more progression in hip function, resulting in improvements in daily activities and, therefore, probably a shorter rehabilitation period. Hip extension can be improved by increasing walking speed and increasing step length [33]. On top of that, the use of walking aids is another important factor to consider. All patients in our study used a walking aid, and as shown in other studies, this affects the dynamic gait pattern and should be taken into consideration when interpreting the data [34].

This study demonstrates that gait performance, specifically step duration and relative peak force, can be measured early after hip fracture surgery in a frail population. Categorizing this performance into quadrants helps to visualize the rehabilitation progress, enabling the identification of patients with weak or strong performances. This categorization allows for a more individualized approach to rehabilitation. Given the expected increase in hip fractures due to an aging population, improvements in postoperative care, such as personalized rehabilitation strategies, are crucial. The use of ambulatory pressure monitoring devices, like the SensiStep, can allow for the early identification of patients at risk for a delayed recovery post surgery.

Several limitations were identified in this study. Variability in the number of steps measured by the device during physiotherapy sessions introduced inconsistencies. The number of patients included in the comparison of surgical methods (proximal femur nail versus hemiarthroplasty) and the 3D gait analysis was small, limiting the generalizability of the findings. Further studies with larger sample sizes in clinical settings are needed to validate these findings. In addition, selection bias is also likely for the 3D gait analysis measurements, as fitter patients predominantly participated. Despite these limitations, the study successfully demonstrates the feasibility of 3D gait analysis in a frail patient population. On top of that, a clinically validated wearable pressure monitoring device showed to be promising in identifying patients with an increased risk for delayed recovery. Future research should focus on individual factors causing this delayed recovery. Furthermore, hip extension after hip fracture surgery was identified as a contributing factor to gait limitations. This is in accordance with other studies but requires further exploration.

## 5. Conclusions

This study demonstrated that elderly patients following hip fracture surgery improve their gait performance during hospitalization, shown by an increased relative peak force and decreased step duration. Similar gait improvements were found for the hemiarthroplasty and the femur nail group. Furthermore, 3D gait analysis showed to be feasible in this frail population and showed significant differences between the affected and healthy leg, especially in hip extension, identifying postoperative rehabilitation needs to improve functionality after hip fracture surgery.

## Figures and Tables

**Table 1:** Patient characteristics of all patients (n = 40), subgroups of patients categorized by proximal femur nail (n = 19) and hemiarthroplasty (n = 10), and patients who underwent a 3D gait analysis (n = 9).

	All Patients	Proximal Femur Nail vs. Hemiarthroplasty		Gait Analysis
		Intramedullary Femur Nail	Hemiarthroplasty	
	N = 40	N = 19	N = 10	N = 9
Gender (female/male)	26/14	12/7	5/5	9/0
Age (years)	79.00 (12.75)	83.00 (16.00)	81.50 (7.00)	77.00 (8.50)
Body height (cm)	167.50 (14.50)	165.00 (13.00)	171.50 (17.00)	167.00 (13.50)
Body weight (kg)	66.50 (15.50)	65.00 (15.00)	77.00 (26.75) *	62.50 (17.50)
BMI (kg/m^2^)	23.70 (4.75)	24.10 (2.80)	24.50 (8.83)	22.60 (4.20)
AHFS	6.00 (4.00)	7.00 (3.00)	8.00 (6.25)	6.00 (3.50)
MMSE	27.00 (4.00)	26.00 (5.00)	26.50 (5.25)	26.00 (4.00)
Length of hospital stay (days)	8.50 (3.00)	9.00 (2.00)	8.00 (3.25)	9.00 (4.00)
Days till first SensiStep measurement	3.00 (2.00)	3.00 (2.00)	2.50 (2.00)	3.00 (1.50)
Days between first and last SensiStep measurement	3.00 (3.00)	4.00 (3.00)	3.50 (1.00)	4.00 (2.50)
Surgical procedure (n):				
Proximal femur nail	19			4
Sliding/dynamic hip screw	2			0
Hemiarthroplasty	10			2
Total hip replacement	5			1
Femoral neck system	4			2

Data are expressed as median (IQR) and absolute numbers (n). BMI: body mass index. AHFS: Almelo Hip Fracture Score. MMSE: mini mental state examination. Proximal femur nail: (Long) gamma nail. * Significant difference (*p* < 0.05) between hemiarthroplasty and proximal femur nail group.

## Data Availability

The data presented in this study are available on request from the corresponding author due to privacy and ethical reasons.

## Abbreviations

The following abbreviations are used in this manuscript:

BMI	Body mass index
QoL	Quality of life
MMSE	Mini-Mental State Examination
MUMC+	Maastricht University Medical Centre+
AHFS	Almelo Hip Fracture Score
SPM	Statistical parametric mapping
PiG	Plug-In Gait
IQR	Interquartile range
SD	Standard deviation
Hz	Hertz
AMTI	Advanced Mechanical Technology, Inc.
SPSS	Statistical Package for the Social Sciences
IBM	International Business Machines
SARS-CoV-2	Severe Acute Respiratory Syndrome Coronavirus 2
3D	Three-dimensional
